# Cardiac Imaging Within Emergency CT Angiography for Acute Stroke Can Detect Atrial Clots

**DOI:** 10.3389/fneur.2019.00349

**Published:** 2019-04-10

**Authors:** Stoyan Popkirov, Uwe Schlegel, Werner Weber, Ilka Kleffner, Jens Altenbernd

**Affiliations:** ^1^Department of Neurology, University Hospital Knappschaftskrankenhaus Bochum, Ruhr University Bochum, Bochum, Germany; ^2^Institute of Radiology, Neuroradiology and Nuclear Medicine, University Hospital Knappschaftskrankenhaus Bochum, Ruhr University Bochum, Bochum, Germany; ^3^Institute of Diagnostic and Interventional Radiology, St. Marien-Hospital Hamm, Hamm, Germany

**Keywords:** angiography, cardioembolic, thrombus, ischemic stroke, atrial fibrillation

## Abstract

Cardiac embolism is presumed to cause a significant portion of cryptogenic strokes. Transesophageal echocardiography may detect intracardiac thrombi, but this remains a rare finding, possibly because remnant clots dissolve spontaneously or following thrombolysis. Cardiac imaging within cerebral CT angiography might offer an alternative method for thrombus detection within hyperacute stroke assessment. In a proof-of-concept study we analyzed records of patients aged ≥ 60 years that presented with suspected stroke and underwent extended cerebral CT angiography as part of their emergency assessment. CT imaging of patients with ischemic stroke or transient ischemic attack (TIA) and atrial fibrillation and of those with embolic strokes of undetermined source (ESUS) was reviewed for intracardiac clots and other cardiac or aortic pathology. Over a period of 3 months 59 patients underwent extended CT angiography for suspected stroke, 44 of whom received a final diagnosis of ischemic stroke or TIA. Of those, 17 had atrial fibrillation, and four fulfilled ESUS criteria. Thrombi were detected within atrial structures on CT angiography in three cases. In two ESUS patients complex atheromatosis of the proximal ascending aorta with irregular and ulcerating plaques was detected. Cardiac imaging within emergency cerebral CT angiography is feasible and can provide valuable diagnostic information in a patient group that might not routinely undergo transesophageal echocardiography. A small change to emergency assessment could potentially uncover cardioembolic pathology in cases that would have remained cryptogenic otherwise.

## Introduction

A significant proportion of embolic strokes of undetermined source (ESUS) are probably cardiogenic, but cardioembolic pathologies can elude standard assessments (1). Transesophageal echocardiography (TEE) is the gold standard for detection of emboligenic abnormalities, but it is not routinely performed in all patients for various reasons. Furthermore, the utility of routine TEE in cryptogenic stroke has been questioned, not least because of a low rate of atrial clot detection (2). A reason for this might be that remnant clots can dissolve shortly after stroke onset and prior to TEE; moreover, systemic thrombolysis can dissolve intracardiac thrombi (3).

An alternative method for detecting left atrial thrombi (and other cardiac pathology) is computed tomography (CT) (4), but ECG-triggered contrast-enhanced cardiac CT would unjustifiably delay thrombolysis and/or thrombectomy. However, relevant cardiac abnormalities, including left atrial clots, can be detected on non-cardiac CT (5, 6). Thus, when cerebral CT angiography (CCTA) is performed to detect large artery occlusion or stenosis within emergency stroke assessment, extending the scan range of the angiogram by several inches to include the heart seems feasible.

Scan range of emergency CCTA for acute stroke in elderly patients included the heart for a period of 3 months at our center. The potential for detecting intracardiac clots in patients with large artery occlusion or transient ischemic attack (TIA) and atrial fibrillation as well as ESUS was investigated.

## Methods

**Figure 1:**
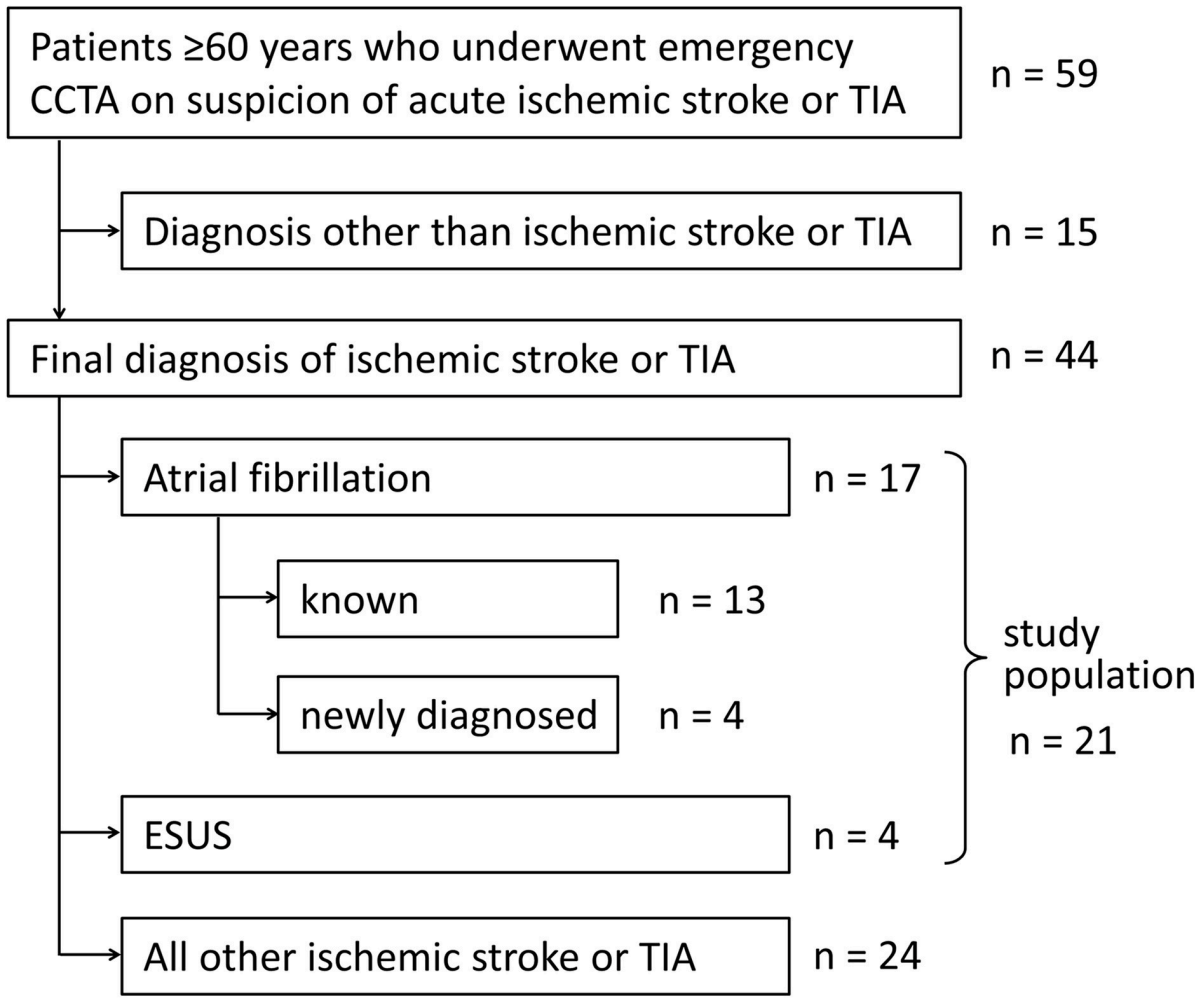
Flow chart showing the selection of the study population.

A proof-of-principle study was conducted to assess the feasibility of performing an extended CCTA that includes the heart as well as its potential to detect atrial clots and other emboligenic pathology. At our center emergency CCTA is routinely performed when large artery occlusion or stenosis is suspected as a cause of acute stroke or TIA according to standard protocol as described elsewhere (7). During the period between 30.04.2018 and 03.08.2018, CT technicians had been instructed to set the lower limit of the scan range not to the aortic arch, but to the diaphragm, for any CCTA that was ordered for suspected stroke in patients ≥ 60 years. To increase the pretest probability of atrial thrombi we only analyzed data of acute stroke or TIA patients who had atrial fibrillation or ESUS (Figure 1). Clinical data was collected retrospectively from electronic records of all consecutive patients during the study period who met inclusion criteria. ESUS diagnosis was assigned to patients who fulfilled all diagnostic criteria and had all required assessments (1). For the purpose of this study, findings from CCTA proximal of the aortic arch were not considered for ESUS status. The effective radiation dose was estimated by multiplying the automatically calculated dose length product (DLP) by a standard conversion factor of 0.014 mSv (mGy·cm)^−1^ (8). This study was approved by the ethics committee of the medical faculty of the Ruhr University Bochum.

## Results

**Figure 2:**
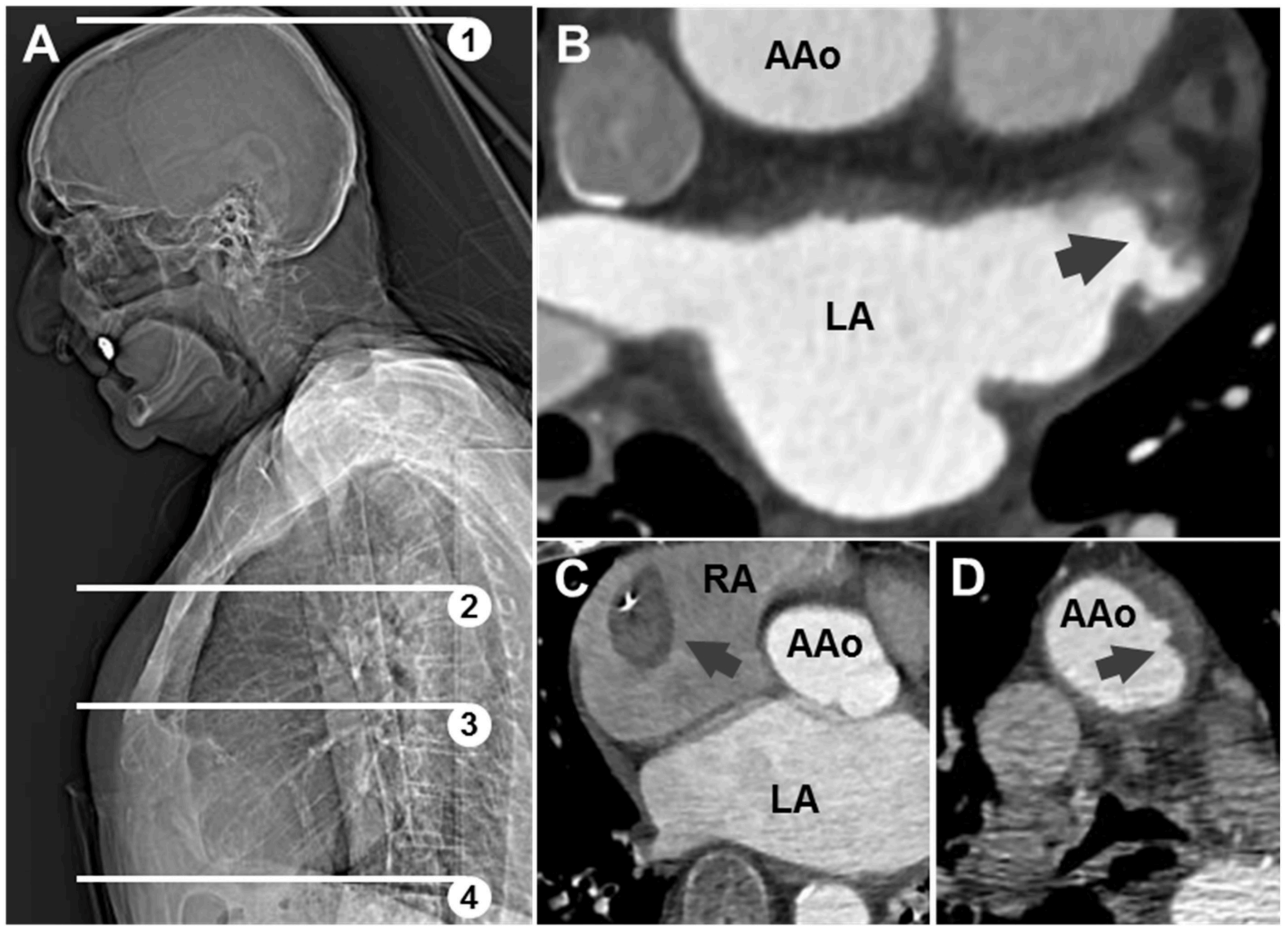
**(A)** Scout-view showing scan range of conventional (1,2) and extended (1–4) angiography; (3) corresponds to panel **(B)**. Axial CT images: **(B)** thrombotic material in appendage of left atrium (LA) in patient 15; **(C)** thrombus around pacemaker lead in right atrium (RA) in patient 17; **(D)** ulcerating plaque in ascending aorta (AAo) in patient with ESUS.

A total of 59 patients underwent emergency CCTA (Figure 2A) for suspected stroke, of which 44 received the final diagnosis ischemic stroke. There were 17 patients who had ischemic stroke or TIA and known or newly diagnosed atrial fibrillation (Table 1; mean age: 77.5 years, standard deviation: 8.4 years; 53% female). As hypothesized, intracardiac thrombi could be visualized: once in an artificially occluded left atrial appendage, once around a pacemaker lead in the right atrium, and once in the left atrial appendage (Figures 2B,C). Four patients were classified as ESUS (Table 2; mean age 80 years, standard deviation 2.7 years; 25% female). Irregular and ulcerating plaques were found in the proximal ascending aorta of two of these patients (Figure 2D).

**Table 1:** Patients with ischemic stroke and atrial fibrillation.

	**Stroke diagnosis**	**Atrial fibrillation**	**Anticoagulation**	**Cardiac pathology in CT angiogram**
1	RICA and RMCA tandem occlusion	Known	Yes (dabigatran 2 × 110 mg)	Coronary atherosclerosis, valvular calcification
2	BA occlusion	Newly diagnosed	No	Coronary atherosclerosis
3	LPCA occlusion	Known	No	Thrombus in occluded left atrial appendage, coronary atherosclerosis
4	Recurring TIA	Known	No (non-adherence)	Coronary atherosclerosis, evidence of cardiac bypass surgery
5	RPCA branch occlusion	Newly diagnosed	No	Coronary atherosclerosis
6	LMCA branch occlusion	Newly diagnosed	No	None
7	RMCA occlusion	Known	Yes (heparin, PTT 35.8 s)^*^	Right atrial enlargement, coronary atherosclerosis
8	RMCA occlusion	Known	No (paused for surgery)	Coronary atherosclerosis, valvular calcification
9	TIA	Known	Yes (phenprocoumon, INR 1.98)	None
10	RICA occlusion	Known	No	Cardiomegaly, coronary atherosclerosis, valvular calcification
11	RMCA branch occlusion	Newly diagnosed	No	Coronary atherosclerosis
12	RMCA infarctions	Known	No (non-adherence)	Coronary atherosclerosis
13	RMCA occlusion	Known	No (non-adherence)	Coronary atherosclerosis
14	RICA occlusion	Known	No	Coronary atherosclerosis, valvular calcification
15	LMCA branch occlusion	Known	No	Thrombus in left atrial appendage, coronary atherosclerosis
16	TIA	Known	Yes (apixaban 2 × 5 mg)	Coronary atherosclerosis, valvular calcification
17	TIA	Known	Yes (apixaban 2 × 2.5 mg) ^†^	Thrombus around pacemaker lead in right atrium, cardiomegaly, coronary atherosclerosis, valvular calcification

*BA, basillary artery; INR, international Normalized Ratio; LMCA, left middle cerebral artery; LPCA, left posterior cerebral artery; PTT, partial thromboplastin time; RICA, right internal carotid artery; RMCA, right middle cerebral artery; RPCA, right posterior cerebral artery; TIA, transient ischemic attack*.

* *Periprocedural heparin during transcatheter mitral valve repair*.

† *Renal clearance ≥ 30 ml/min, serum creatinine < 1,5 mg/dl, and weight > 60 kg; half-dose due to history of bleeding, presumably insufficient/ineffective*.

**Table 2:** Patients with embolic stroke of undetermined source (ESUS)^*^.

	**Stroke diagnosis**	**Cardiac pathology in CT angiogram**	**Echocardiography**
18	RMCA branch occlusion	Atheromatosis with irregular plaques in proximal ascending aorta, coronary atherosclerosis, valvular calcification	TTE: mildly dilated left atrium
			TEE: ulcerating aortic plaques with floating parts, mildly dilated left atrium, persistent foramen ovale
19	RPCA occlusion	Atheromatosis with ulcerating plaques in proximal ascending aorta, coronary atherosclerosis, valvular calcification	TTE: mildly dilated left atrium
20	LMCA occlusion	Coronary atherosclerosis, valvular calcification	TTE: mildly dilated left atrium, aortic sclerosis
21	LMCA branch occlusion	Coronary atherosclerosis	TTE and TEE: mildly dilated left atrium

*LMCA, left middle cerebral artery; RMCA, right middle cerebral artery; RPCA, right posterior cerebral artery; TEE, transesophageal echocardiography; TTE, transthoracic echocardiogram*.

* *For the purpose of this study findings first identified on CT angiography proximal of the aortic arch were disregarded for ESUS criteria*.

Median DLP for all 44 ischemic stroke patients was 358 mGy·cm (interquartile range, 305–549 mGy·cm); corresponding radiation doses were thus 5.01 mSv (interquartile range, 4.27–7.69 mSv).

## Discussion

Our results demonstrate that co-imaging of cardiac structures within emergency CCTA in elderly patients with ischemic stroke or TIA and atrial fibrillation or ESUS can indeed visualize intracardiac clots before thrombolytic treatment, as well as other emboligenic pathologies such as ulcerating plaques of the ascending aorta.

The main advantage of cardiac imaging within routine CCTA compared to dedicated ECG-gated cardiac CT is the minimal delay of only a few extra seconds, and the sparing of additional contrast agent. A potential disadvantage is lower image quality due to heart movement artifacts and variable contrast filling since bolus timing is optimized for cerebral arterial visualization (9). However, studies suggest that ECG-gating does not markedly improve image quality of the ascending aorta (10) or the left atrium (11). Image quality in this study was adequate in most cases, though larger samples with control imaging would be needed to quantify differences. While TEE remains the gold standard for intracardiac clot detection, it requires specialized expertise and is time-consuming. Performing it in an emergency setting before thrombolysis would introduce an unjustifiable time delay.

The additional radiation exposure during cardiac imaging added to CCTA has to be considered. In our study the median DLP and estimated radiation dose for the entire extended CCTA were less than average for cardiac CT angiography (8). The lifetime risk of radiation-induced fatal cancer for this procedure is <0.05%, added to an average background risk of 21% (12). Although our study design did not allow calculating the added radiation exposure in extended CCTA compared to routine CCTA, the added risk would naturally be even lower. Especially in an elderly population, radiation-associated risks thus can be considered negligible. Moreover, comprehensive risk analysis should also consider periprocedural risks of TEE [0.02% for serious complications (2)] and the risk of cryptogenic stroke recurrence (3–6%/year) (1).

Our study design does not allow estimations of the diagnostic yield of extended CCTA, pre-treatment prevalence of remnant left atrial clots, or direct comparisons with other methods. However, our findings serve as a proof of principle: intracardiac clots can be detected on extended emergency CCTA before they are potentially dissolved by thrombolysis or flushed into the circulation. We looked at patients with known atrial fibrillation to increase pretest probability for cardiac thrombi. However, the biggest clinical benefit of this method would be expected in patients with cryptogenic stroke, where detecting a cardiac thrombus has profound implications for acute treatment (early anticoagulation, follow-up imaging) and secondary prevention (long-term anticoagulation). Prospective multi-center studies should explore this idea in the future. In the long run, a “one-stop shop” multimodal CT program could be developed to optimize emergency stroke diagnostics.

## Data Availability

The datasets generated for this study are available on request to the corresponding author.

## Ethics Statement

The study protocol was approved by the ethics committee of the Medical Faculty of Ruhr University Bochum. This was a retrospective study based on routine clinical data, so written informed consent was not acquired.

## Author Contributions

SP had the idea for the study and served as principal investigator, performed data analysis and wrote the first draft of the manuscript. US and WW contributed to the analysis. IK and JA contributed to clinical and radiological data acquisition and analysis. All authors revised and approved the final manuscript.

### Conflict of Interest Statement

The authors declare that the research was conducted in the absence of any commercial or financial relationships that could be construed as a potential conflict of interest.
